# The Novel Cyclophilin Inhibitor CPI-431-32 Concurrently Blocks HCV and HIV-1 Infections via a Similar Mechanism of Action

**DOI:** 10.1371/journal.pone.0134707

**Published:** 2015-08-11

**Authors:** Philippe A. Gallay, Michael D. Bobardt, Udayan Chatterji, Daniel J. Trepanier, Daren Ure, Cosme Ordonez, Robert Foster

**Affiliations:** 1 Department of Immunology & Microbial Science, The Scripps Research Institute, La Jolla, California, United States of America; 2 Ciclofilin Pharmaceuticals Inc., San Diego, California, United States of America; Rosalind Franklin University of Medicine and Science, UNITED STATES

## Abstract

HCV-related liver disease is the main cause of morbidity and mortality of HCV/HIV-1 co-infected patients. Despite the recent advent of anti-HCV direct acting antivirals (DAAs), the treatment of HCV/HIV-1 co-infected patients remains a challenge, as these patients are refractory to most therapies and develop liver fibrosis, cirrhosis and liver cancer more often than HCV mono-infected patients. Until the present study, there was no suitable *in vitro* assay to test the inhibitory activity of drugs on HCV/HIV-1 co-infection. Here we developed a novel *in vitro* “co-infection” model where HCV and HIV-1 concurrently replicate in their respective main host target cells—human hepatocytes and CD4+ T-lymphocytes. Using this co-culture model, we demonstrate that cyclophilin inhibitors (CypI), including a novel cyclosporin A (CsA) analog, CPI-431-32, simultaneously inhibits replication of both HCV and HIV-1 when added pre- and post-infection. In contrast, the HIV-1 protease inhibitor nelfinavir or the HCV NS5A inhibitor daclatasvir only blocks the replication of a single virus in the “co-infection” system. CPI-431-32 efficiently inhibits HCV and HIV-1 variants, which are normally resistant to DAAs. CPI-431-32 is slightly, but consistently more efficacious than the most advanced clinically tested CypI—alisporivir (ALV)—at interrupting an established HCV/HIV-1 co-infection. The superior antiviral efficacy of CPI-431-32 over ALV correlates with its higher potency inhibition of cyclophilin A (CypA) isomerase activity and at preventing HCV NS5A-CypA and HIV-1 capsid-CypA interactions known to be vital for replication of the respective viruses. Moreover, we obtained evidence that CPI-431-32 prevents the cloaking of both the HIV-1 and HCV genomes from cellular sensors. Based on these results, CPI-431-32 has the potential, as a single agent or in combination with DAAs, to inhibit both HCV and HIV-1 infections.

## Introduction

Since HCV and HIV share the same routes of transmission, co-infection is a frequent event, occurring in 5–10 million individuals worldwide [1–2]. The current primary route of exposure of both viruses is through contaminated needles. It is estimated that 50%-90% of injection drug users are infected with HCV due to the high efficiency of HCV transmission via percutaneous blood exposure [3–10]. The negative impact of HIV-1 infection on hepatitis C is well known [11–13]. HIV-1/HCV co-infection is associated with higher HCV viral load, persistent HCV viremia, reduced response to IFN alpha-based HCV treatment, and accelerated and more aggressive liver disease. Higher HCV RNA levels and chronic HCV infection in HIV-1-infected patients are thought to be related to diminution of CD4 and CD8 T-cell responses to HCV infection [14–16]. HIV-1-derived proteins such as tat and gp120 may mediate a hepatic cytokine milieu via binding to hepatocytes, stellate cells, and immune cell populations resident in the liver [17].

Despite highly active antiretroviral therapy (HAART), there is an increased risk of hepatitis/liver-related deaths among co-infected drug users compared to HCV-mono-infected drug users [18]. Moreover, HCV-mediated accelerated liver disease is thought to be the main cause of the mortality in HIV-1/HCV co-infected patients [19]. One strategy to address these problems is to identify drugs that concurrently diminish infection and replication of both HCV and HIV-1. Since CypI exhibit antiviral activities against both HIV-1 and HCV individually, we asked in this study whether CypI could inhibit HCV and HIV-1 in the context of co-infection. Indeed, HIV-1 was found to rely on CypA to optimally replicate in human cells and found to be sensitive to CypI such as CsA and non-immunosuppressive CsA derivates [20–25]. Similarly, HCV was found to absolutely require CypA to replicate both *in vitro* and *in vivo* and that CsA, CsA derivates, sanglifehrins and sanglifehrin derivates block its replication [26–44].

## Materials and Methods

### Drugs

The HCV NS5Ai daclatasvir (Daklinza) (Bristol Myers Squibb) and the HIV-1 protease inhibitor nelfinavir were obtained from MedChemexpress (Princeton, NJ 08540, USA). The CypIs ALV and CPI-431-32 were obtained from WuXi AppTec and Ciclofilin Pharmaceuticals Inc, respectively, whereas CsA was obtained from Sigma-Aldrich, St-Louis, MO, USA).

### Cells and viruses

#### HIV-1

The HIV-1 target cells—blood-derived CD4^+^ T-lymphocytes—were isolated as described previously [45]. The Scripps Research Institute Normal Blood Donor Service (TSRI NBDS) provides investigators at TSRI who have Human Subjects Committee-approved protocols with a source of normal blood for their research. Donors are assured of a controlled clinical setting for their blood to be drawn by licensed phlebotomists, and investigators are assured that the donors whose specimens they obtain through the service have been screened upon entry into the program and annually thereafter for a CBC, Hepatitis B and C and HIV. Hemoglobin determinations at every donation protect the donor from phlebotomy-induced anemia. The donor pool also provides investigators with a mix of gender and minority subjects, and recruitment is ongoing for underrepresented minorities. At the present time, the NBDS has 320 active normal blood donors enrolled. Use of the Normal Blood Donor Service is considered “human subjects” research and each investigator who wants to use the service must submit a protocol to the IRB for review and approval. Dr. Gallay has current IRB approval for the human blood obtained for research described in this manuscript. Protocol No: IRB-15-6552. Full name of the IRB is "Scripps IRB". The IRB entitled "Evaluation of the antiviral activities of CPI-431-32 on the HBV/HIV-1 co-infection" specifically approved the use of blood donor in this study. Consent from blood donors was obtained verbally. The authors have no access to identifying donor information for these samples. The primary HIV-1 isolate JR-CSF and the lab-adapted NL4.3 viruses were obtained through the NIH AIDS Research and Reference Reagent Program. JR-CSF was amplified in PBMC (5 x 10^6^ cells), and infectious particles collected 2–3 weeks post-infection and quantified by HIV-1 p24 ELISA (HIV1: Alliance HIV-1 p24 ELISA Kit from PerkinElmer).

#### HCV

The HCV target cells—human hepatoma Huh7.5.1 cells—were provided by F. Chisari and grown as described previously [46]. The HCV virus JFH-1 was generated by electroporation of Huh7.5.1 cells with full genomic luciferase reporter replicon Luc-Neo-JFH-1 previously created [47–50]. JFH-1 infectious particles were collected 20–30 post-electroporation, concentrated ~10-100-fold, and quantified by HCV core ELISA (Ortho HCV antigen ELISA kit; Ortho Clinical Diagnostics, Waco Chemicals, Inc. or HCV Core ELISA, XpressBio).

### Mono-infection and co-infection systems

#### Mono-infections

HIV-1: Activated CD4+ T-lymphocytes (500,000) were exposed to 1 ng of p24 of JR-CSF for 3 h, washed and cultured for 3 weeks in flasks (triplicates). Aliquots of cell culture supernatants were collected every 3 days, filtered to remove cell debris and frozen until HIV-1 quantification by p24 ELISA. HCV: Huh7.5.1 cells (500,000) were exposed to 1 ng of core of JFH-1 3 h, washed and cultured for 3 weeks in flasks (triplicates). Aliquots of cell culture supernatants were collected every 3 days, filtered to remove cell debris and frozen until HCV quantification by core ELISA.

#### Co-infections/Concurrent infections

CD4+ T-lymphocytes and Huh7.5.1 cells uninfected or infected for 3 h as described above were mixed in a single flask and HIV-1 and HCV replication monitored over a period of 3 weeks as described above.

### Drug-resistant HIV-1 and HCV analyses

#### Drug-resistant HIV-1 studies

the reverse transcriptase inhibitor-resistant HIV-1 variant (nevirapine-resistant N119) [51], the protease inhibitor-resistant HIV-1 variant (saquinavir-resistant) [52], and the naturally occurring primary ALV-resistant variant CMU08 (contributed by Kenrad Nelson) were obtained from the AIDS Research and Reference Reagent Program. Lab-adapted wild-type NL4.3 and the ALV-resistant V86P/H87Q/I91V/M96I NL4.3 capsid mutant variant were described previously [53]. For infection scoring, TZM-bl target cells (contributed by J. C. Kappes, X. Wu, and Tranzyme, Inc.) obtained through the AIDS Research and Reference Reagent Program were exposed to wild-type or drug-resistant HIV-1 variants (1 ng of p24) together with increasing concentrations of DMSO or CypI. HIV-1 infection was quantified 48 h post-infection by measuring β-galactosidase activity in cell lysates.

#### Drug-resistant HCV studies

The GT1b subgenomic firefly luciferase reporter replicon pFK-I389/NS3–3 was provided by R. Bartenschlager. The GT1B subgenomic NS3 (R155Q/A156T), NS5A (L31V, D320E/Y321N, S328P and P313A/P318A), and NS5B (S282T) mutants were created as previously [54]. Half-maximal effective concentration (EC_50_) measurements were conducted as previously [29].

### CypA-HCV NS5A and CypA-HIV-1 capsid interaction analyses

Recombinant GST-CypA, full-length HCV NS5A Con1 (pET-Ub-NS5A Con1-His) and HIV-1 capsid NL4.3 (pET-capsid-His) were expressed and purified as described previously [55]. Inhibition of CypA-HCV NS5A and CypA-HIV-1 capsid interactions by the CypI CPI-432-31 were conducted as we described previously [55].

### CypA isomerase inhibition assay

Inhibition of CypA isomerase activity was assessed using the α-chymotrypsin-coupled assay adapted to a 96-well plate format [56–57]. Human recombinant CypA (Atgen) was dissolved to 10 nM in isomerase buffer (50 mM Hepes, 100 mM NaCl, 1 mg/ml bovine serum albumin, 1 mg/ml α-chymotrypsin; pH 8). Succinyl-AAPF-pNA peptide substrate (Sigma) was dissolved to 3.2 mM in dried LiCl/trifluoroethanol. Each test compound was prepared at 10 concentrations in DMSO, then diluted into CypA-isomerase buffer to 0.05–1000 nM (reaction mix). All solutions were equilibrated, and reactions conducted at 5°C. Reactions were initiated by mixing 95 μL reaction mix with 5 μL peptide preloaded in multiple wells of 96-well plates and measuring OD405 nm in each well at 6-sec intervals for 6 min using a BMG Polarstar Galaxy plate reader. Data were fitted with Graphpad Prism 6.0 to obtain first-order rate constants. Enzyme-catalyzed rate constants were calculated by subtracting the rate constant from uncatalyzed reactions (no CypA), and the catalytic rate constants plotted as a function of inhibitor concentration to obtain IC_50_s.

#### HIV-1 quantitative real-time PCR

Early and late NL4.3 reverse transcripts were quantified as described previously [58].

#### HIV-1 nuclear import analysis

CD4+ HeLa cells (10^7^ cells) were infected with NL4.3 (1 μg of p24) derived from 293T-transfected cells in the presence or absence of CPI-431-32 (2 μM). Six hours post-infection, cells were fractionated using the Thermo Scientific NE-PER nuclear and cytoplasmic extraction kit (Life Technologies). Cytoplasmic and nuclear extracts were analyzed by Western blotting using anti-HIV-1 integrase (Abcam), anti-CypA (Abcam) and anti-histone H2 (Abcam) antibodies.

#### HCV reverse transcription-quantitative PCR analysis

HCV RNA levels were quantified via reverse transcription-quantitative PCR (RT-qPCR) as we described previously [59] and the results presented as genome equivalents (GE) per microgram of total RNA.

#### HCV infectivity analysis

HCV infectivity was quantified by indirect immunofluorescence using anti-NS5A IgG as we described previously [59] and results expressed as focus-forming units (FFU) per milliliter of supernatant, determined by the average number of NS5A-positive foci detected at the highest dilutions.

#### Double membrane vesicle (DMVs) formation analysis

HCV-mediated DMV formation studies were conducted as we described recently [60] using the pTM1(NS3-NS5B) plasmid and the T7/Huh7.5.1 cells developed by the Tai lab [61].

## Results

### CPI-431-32 efficiently inhibits HIV-1 and HCV mono-infection

**Fig 1:**
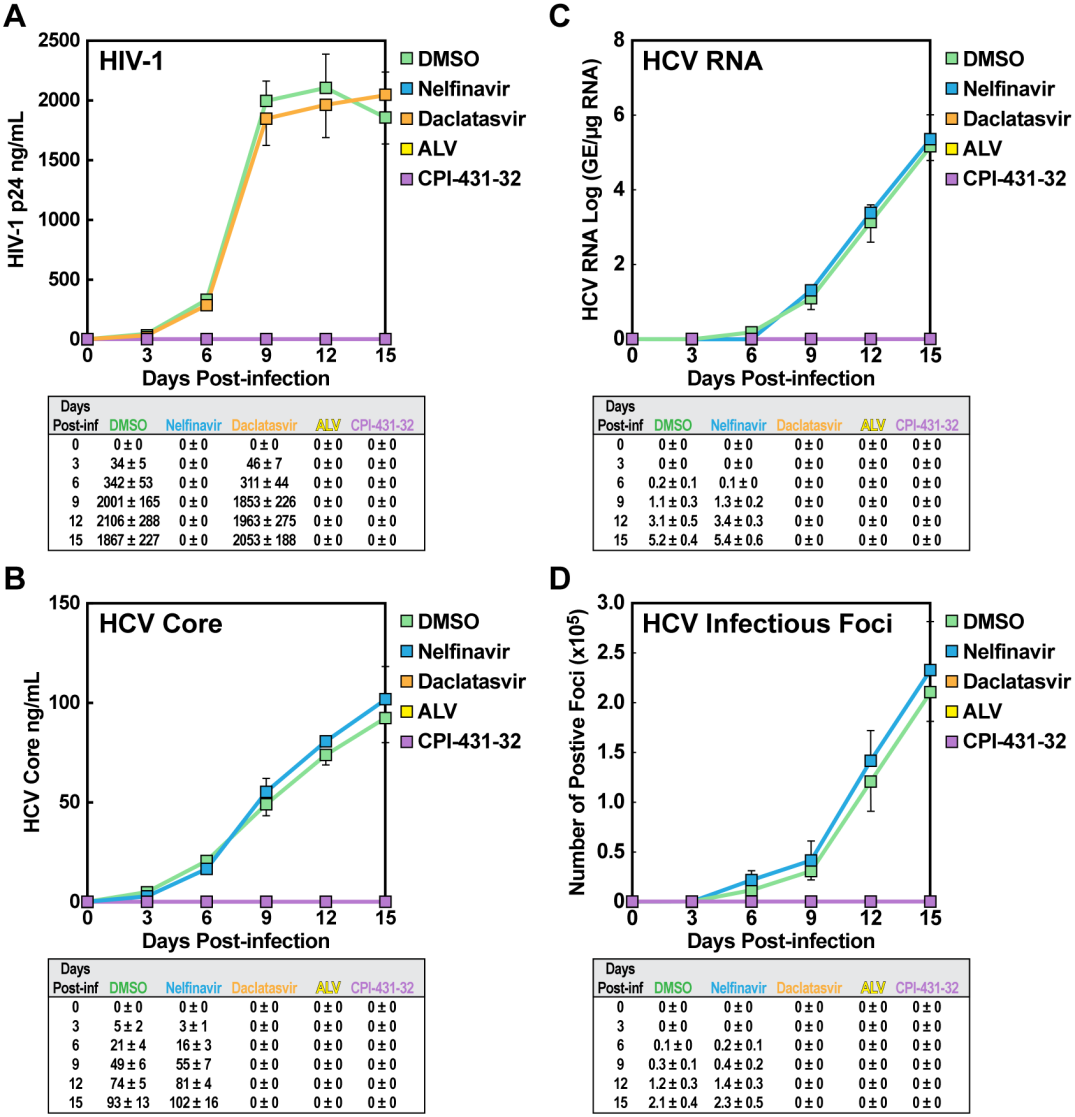
CPI-431-32 blocks both HIV-1 and HCV mono-infection. CD4+ T-lymphocytes (0.5 x 10^6^ cells) (triplicates) were exposed to HIV-1 JR-CSF (1 ng of p24) together with DMSO, the HIV-1 protease inhibitor nelfinavir, the HCV NS5A inhibitor daclatasvir and the two CypI ALV and CPI-431-32. Virus and drugs were washed away after 3 h and HIV-1 replication monitored for a period of 2 weeks every 3 days by quantifying amounts of virus in the cell culture supernatant by p24 ELISA (**A**). Same as A, except Huh7.5.1 hepatoma cells (0.5 x 10^6^ cells) (triplicates) were exposed to HCV JFH-1 (1 ng of core) and replication monitored by ELISA for HCV core levels in supernatants (**B**), by RT-qPCR for HCV RNA levels in supernatants (**C**) or by colony forming assay for HCV infectious units in supernatants (**D**). Results are representative of two independent experiments.

To determine whether CypI represent effective drug candidates for HIV-1/HCV co-infection, we first verified the antiviral activity of two CypI in HIV-1 and HCV mono-infections—a novel nonimmunosuppressive, CsA analog, CPI-431-32, and the most clinically advanced CypI, Alisporivir (ALV). To study HIV-1 mono-infection, isolated and activated CD4+ T-lymphocytes (pool of 3 blood donors) were exposed to the prototype primary R5 isolate JR-CSF in combination with the following drug treatments: i) DMSO vehicle; ii) HIV-1 protease inhibitor nelfinavir as positive control; iii) HCV NS5A inhibitor daclatasvir as negative control; and iv) CypI—either CPI-432-31 or ALV. Cells were exposed first to drug treatment, followed immediately by virus addition. After three hours, cells were washed and maintained for two weeks without new drug addition. Aliquots of cell culture supernatant aliquots were collected every three days for measurement of the HIV-1 capsid protein, p24. We observed a peak of viral growth seven days post-infection followed by a plateau likely due to widespread infection of cells influencing their cell division and/or viability (Fig 1A). Values and standard deviations are also presented. As expected, the specific HIV-1 inhibitor, nelfinavir, totally blocked replication, whereas the specific HCV inhibitor, daclastasvir had no effect (Fig 1A). Both CypI—ALV and CPI-431-32—efficiently blocked viral growth (Fig 1A). We observed similar results in two independent experiments using each a pool of 3 blood donors as source of CD4+ T-lymphocytes (data now shown).

We conducted a similar experiment for HCV. Human hepatoma Huh7.5.1 cells were exposed to the same panel of drugs immediately followed by addition of the infectious HCV virus JFH-1 and viral replication quantified by HCV core ELISA (Fig 1B). Levels of HCV core in supernatants correlated well with those of HCV RNA (Fig 1C) or infectious HCV particles (Fig 1D). As anticipated, daclastasvir fully blocked HCV replication whereas nelfinavir had no effect (Fig 1B). Both CypI also efficiently blocked HCV replication (Fig 1B). Note that at high viral inoculi, we consistently observed that CypIs inhibit HCV replication more efficiently than HIV-1 replication, suggesting that HCV relies more on CypA than HIV-1 to replicate in human cells (data not shown). Together these data indicate that CypIsrepresent attractive drug candidates for the treatment of HIV-1/HCV co-infection.

### CPI-431-32 effectively inhibits HIV-1/HCV co-infection

**Fig 2:**
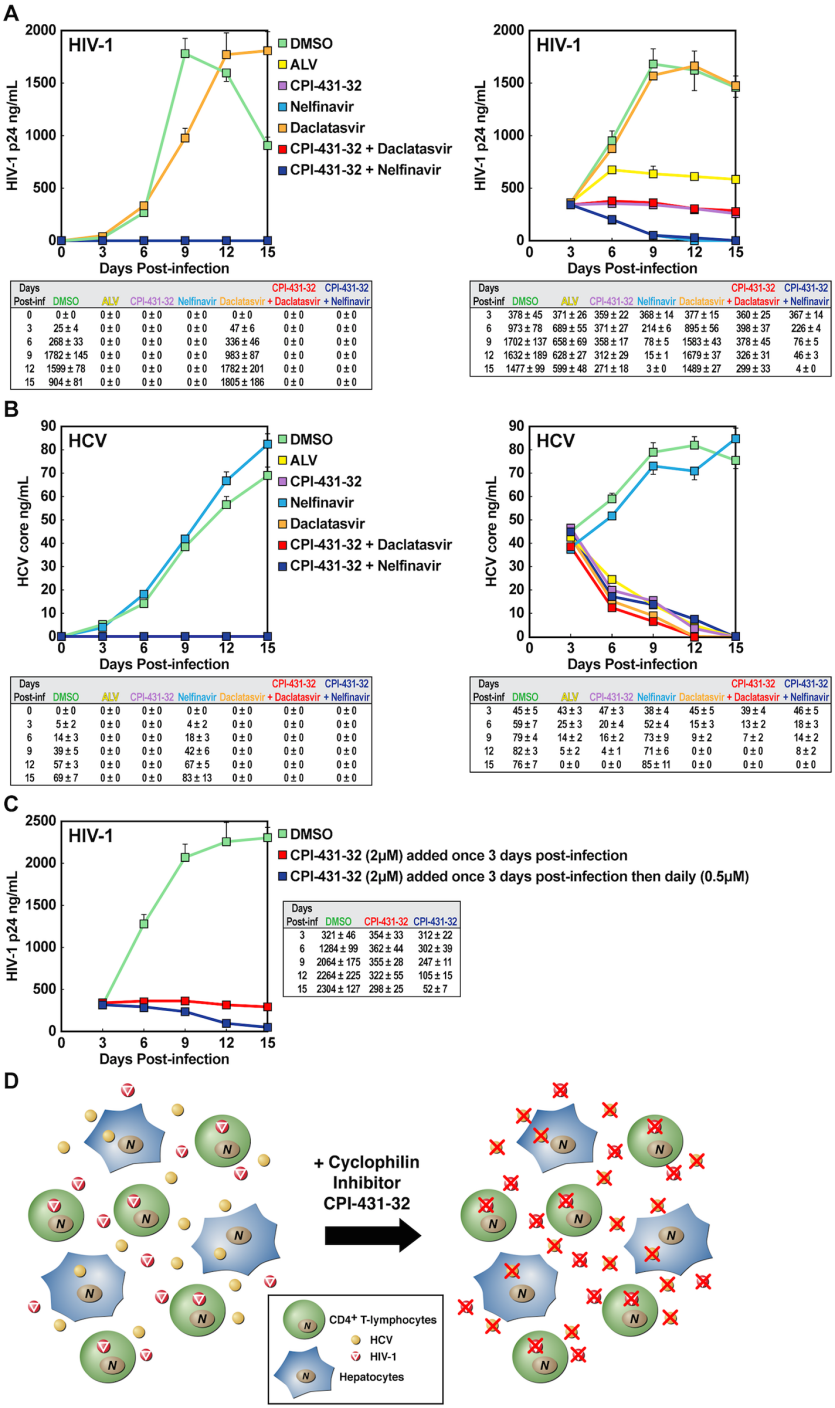
CPI-431-32 simultaneously blocks HIV-1 and HCV infections. CD4+ T-lymphocytes (**A**) and Huh7.5.1 cells (**B**) (0.5 x 10^6^ cells) (triplicates) were exposed to HIV-1 JR-CSF and HCV JFH-1. Drugs (2 μM) were added 3 h pre-viral exposure (top panels) or 3 days post-viral exposure (bottom panels). Viral replications were monitored by HIV-1 p24 and HCV core ELISAs. **C.** CD4+ T-lymphocytes (0.5 x 10^6^ cells) (triplicates) were exposed to HIV-1 JR-CSF. Drugs were initially added 3 days post-viral exposure (2 μM) and then daily (0.5 μM). Viral replications were monitored by HIV-1 p24 and HCV core ELISAs. Results are representative of two independent experiments. **D.** Model for the HIV-1/HCV co-culture system.

After demonstrating that CypI blocked HIV-1 and HCV mono-infections, we asked whether they could inhibit concurrent infections in a co-culture system. HIV-1 target cells—CD4+ T-lymphocytes—and HCV target cells—hepatoma cells—were placed in a single chamber (flask), exposed to both HIV-1 and HCV, and viral replication monitored over time by p24 capsid and core ELISAs, respectively. When drug treatments were begun 3 h before virus addition, CPI-431-32, ALV, and the HIV-1 inhibitor, nelfinavir, totally prevented HIV-1 replication (Fig 2A, left panel). As expected, CPI-431-32/daclatasvir and CPI-431-32/nelfinavir drug combinations also completely prevented HIV-1 replication whereas DMSO or daclastasvir alone did not (Fig 2A, left panel). HCV replication was completely prevented by CPI-431-32, ALV, and the HCV NS5A inhibitor, daclastasvir, and by two-drug combinations thereof, whereas DMSO and the HIV-1 inhibitor, nelfinavir, did not (Fig 2B, left panel). Together these data indicate that CypI, possess the ability to simultaneously block HIV-1 and HCV replication in an *in vitro* co-culture system.

To address whether CypI could also block established infections, co-culture experiments were run in which drug treatments were begun 3 days post-infection. In the absence of drug, both HIV-1 and HCV replicated robustly for approximately 9 days (Fig 2A and 2B, right panels). A single dose of CPI-431-32 added 3 days post-infection halted HIV-1 replication and produced a slight, gradual decline in virus release over 2 weeks. ALV produced a similar response except that a small degree of replication occurred within the first 3 days of addition to the culture (Fig 2A and 2B, right panels). HCV was more sensitive than HIV-1 to CypI. CPI-431-32 and ALV immediately and gradually caused a decline in HCV levels over the course of the experiment. Nelfinavir and daclatasvir completely eradicated the respective replication of HIV-1 and HCV (Fig 2A and 2B, right panels). We showed above that a single addition of 2 μM of CPI-431-32 three days post-HIV-1 infection only partially suppressed HIV-1 replication. We thus asked whether adding daily CPI-431-32 after the initial drug exposure three days post-infection would more profoundly inhibit HIV-1 replication. We chose a daily CPI-431-32 dose of 0.5 μM. The repeated addition of CPI-431-32 almost totally repressed HIV-1 replication after fifteen days of drug treatment (Fig 2C) without apparent cellular toxicity as monitored by lactate dehydrogenase (LDH) release into the supernatant (data not shown). Fig 2D displays the HIV-1/HCV co-culture/co-infection experimental system.

### CPI-431-32 shows efficacy against drug-resistant HIV-1 and HCV variants

We then tested the efficacy of CPI-431-32 and ALV against a panel of drug-resistant HIV-1 and HCV variants. Specifically, we measured CPI-431-32 EC_50_ on a reverse transcriptase inhibitor-resistant HIV-1 variant (nevirapine-resistant) [51], a protease inhibitor-resistant HIV-1 variant (saquinavir-resistant) [52], and two partially ALV-resistant HIV-1 variants—a lab-adapted resistant variant (V86P/H87Q/I91V/M96I capsid mutant) and a naturally occurring primary partially resistant variant (CMU08) [53]. For HIV-1 infection, CD4/CXCR4/CCR5+ TZM *β-galactosidase* reporter target cells were exposed to wild-type or drug-resistant HIV-1 variants (1 ng of p24) together with increasing concentrations of DMSO or CypI—CsA, ALV, or CPI-431-32. HIV-1 infection was quantified 48 h post-infection by measuring β-galactosidase activity in cell lysates. All CypI—CsA, ALV and CPI-431-32—efficiently inhibited the infectivity of wild-type (0.95, 0.12 and 0.05 μM, respectively), protease-resistant (1.12, 0.16 and 0.07 μM, respectively) and reverse transcriptase-resistant (0.98, 0.1 and 0.03 μM, respectively) viruses (Table 1). We consistently found the following relative degrees of inhibition—CPI-431-32>ALV>CsA (Table 1). CPI-431-32 remained relatively potent against the two ALV-resistant viruses—the V86P/H87Q/I91V/M96I capsid mutant variant and the primary isolate CMU08–0.9 and 2.1 μM, respectively (Table 1).

**Table 1:** CPI-431-32 inhibits the infection and replication of a panel of drug-resistant HIV-1 and HCV variants. For HIV-1 infection, TZM cells were exposed to wild-type or HIV-1 drug-resistant variants together with increasing concentrations of DMSO, CsA, ALV or CPI-431-32. HIV-1 infection was quantified 48 h post-infection by measuring β-galactosidase activity in cell lysates. For HCV replication, Huh7.5 cells expressing wild-type or Luc-Con1 HCV drug-resistant variant replicons were exposed to increasing concentrations of DMSO, CsA, ALV or CPI-431-32. HCV replication was quantified 72 h post-infection by measuring luciferase activity in cell lysates. Data are expressed as EC_50_ that is the drug concentration necessary for the inhibition of HIV-1 infection (drug concentration expressed in μM) and HCV replication (drug concentration expressed in nM) in the absence of the drug by half.

Viruses	CsA	ALV	CPI-431-32
HIV-1 wild-type (NL4.3)	0.95 +/- 0.08	0.12 +/- 0.03	0.05 +/- 0.01
HIV-1 reverse transcriptase (nevirapine)-resistant	0.98 +/- 0.06	0.1 +/- 0.02	0.03 +/- 0.01
HIV-1 protease (saquinavir)-resistant	1.12 +/- 0.09	0.16 +/- 0.02	0.07 +/- 0.02
HIV-1 ALV-resistant (NL4.3 V86P/H87Q/I91V/M96I)	7.9 +/- 0.08	2.3 +/- 0.3	0.9 +/- 0.1
HIV-1 ALV-resistant (CMU8)	9.8 +/- 0.3	5.6 +/- 0.4	2.1 +/- 0.1
HCV wild-type (Con1b)	102.4 +/- 9.2	16.6 +/- 0.8	9.3 +/- 0.4
HCV NS3-resistant (R155Q/A156T)	99.1 +/- 7.3	17.7 +/- 1.9	11.3 +/- 0.2
HCV NS5A-resistant (L31V)	112.3 +/- 8.4	16.5 +/- 2.1	8.9 +/-0.4
HCV NS5B-resistant (S282T)	108.1 +/- 7.8	15.6 +/- 2.4	8.3 +/- 0.4
HCV ALV-resistant (D320E/Y321N)	374.1 +/- 28.4	44.2 +/- 5.8	26.5 +/- 3.2
HCV CsA-sensitive (S328P)	27.3 +/- 1.3	5.6 +/- 1.1	5.1 +/- 0.3
HCV CsA-sensitive (P313A/P318A)	22.4 +/- 3.1	5.2 +/- 0.6	4.7 +/- 0.2

+/- values represent the standard errors of triplicates. Results are representative of two independent experiments.

We then examined whether CPI-431-32 is effective against HCV variants, which are resistant to direct-acting antivirals (DAA). We took advantage of the DAA-resistant HCV genotype 1b Con1 replicons that we previously generated [53]: the NS3 inhibitor-resistant R155Q/A156T NS3, the NS5A inhibitor-resistant L31V NS5A replicon, and the NS5B inhibitor-resistant S282T NS5B replicons. We also examined the effect of CPI-431-32 on the ALV-resistant D320E/Y321N HCV replicon as well as the CsA-sensitive S328P and P313A/P318A HCV replicons [62–66]. We compared the efficacy of the three CypI—CsA, ALV and CPI-431-32. We found that CPI-431-32 is highly efficacious against all DAA-resistant HCV variants and against the partially ALV-resistant and CsA-sensitive variants (Table 1). EC_50_ HCV data are expressed in nM whereas EC_50_ HIV-1 data are expressed in μM. Our findings that HIV-1 and HCV EC_50_ for CIP-431-32 and ALV are very similar, suggest a cross-resistance between ALV and CIP-431-32. Together these data further suggest that CPI-431-32 represents an attractive antiviral for HCV/HIV-1 co-infection.

### CPI-431-32 efficiently inhibits both CypA-HIV-1 capsid and CypA-HCV NS5A interactions

CypI are thought to inhibit the infection of HIV-1 and HCV mostly by preventing CypA-HIV-1 capsid and CypA-HCV NS5A interactions, respectively [55]. The potency of CypI at inhibiting these interactions was determined using an ELISA assay previously developed in our laboratory [55]. For analyzing CypA-HCV NS5A interactions, recombinant NS5A-His was added to GST-CypA-coated wells for 16 h at 4°C in the absence or presence of CypI, followed by detection of captured NS5A-His with anti-His antibodies. A similar assay was used for CypA-HIV-1 interactions except with recombinant His-tagged HIV-1 capsid. For the inhibition of CypA-HCV NS5A interactions, we calculated IC_50_s of 1.35 ± 0.32, 0.42 ± 0.05 and 0.18 ± 0.03 μM for CsA, ALV and CPI-431-32, respectively (Table 2). For the inhibition of CypA-HIV-1 capsid interactions, we calculated IC_50_s of 1.30 ± 0.22, 0.41 ± 0.05 and 0.21 ± 0.01 μM for CsA, ALV and CPI-431-32, respectively (Table 2). The higher potency of CPI-431-32 over CsA and ALV at blocking CypA-HCV NS5A and CypA-HIV-1 capsid interactions thus may explain its superior antiviral efficacy towards HIV-1 and HCV infections.

**Table 2:** CypA-HCV NS5A and CypA-HIV-1 Capsid Complex Inhibition Analyses. Plates were coated with GST-CypA and blocked as we described previously [30]. Recombinant NS5A-His or capsid-His (1 ng/ml) was added to wells in binding buffer (20 mM Tris [pH 7.9], 0.5 M NaCl, 10% glycerol, 10 mM DTT, and 1% NP-40) together with increasing concentrations of DMSO, CsA, ALV or CPI-431-32 for 16 h at 4°C. Captured NS5A-His or capsid-His was subsequently detected using mouse anti-His antibodies and rabbit anti-mouse horseradish peroxidase phosphatase (HRP)-conjugated antibodies as we described previously [30]. After adding the OPD substrate, plates were read on a plate reader at 490 nm. Data are expressed as the concentration (IC_50_ nM) of CypI necessary to inhibit 50% of NS5A or capsid binding to CypA in the absence of drug.

	CsA	ALV	CPI-431-32
CypA-HIV-1 capsid complex	1.32 +/- 0.21	0.42 +/- 0.11	0.23 +/- 0.01
CypA-HCV NS5A complex	1.35 +/- 0.32	0.42 +/- 0.05	0.18 +/- 0.03

+/- values represent the standard errors of triplicates. Data are representative of two independent ELISA experiments.

### CPI-431-32 inhibits the isomerase activity of CypA more efficiently than CsA and ALV

**Fig 3:**
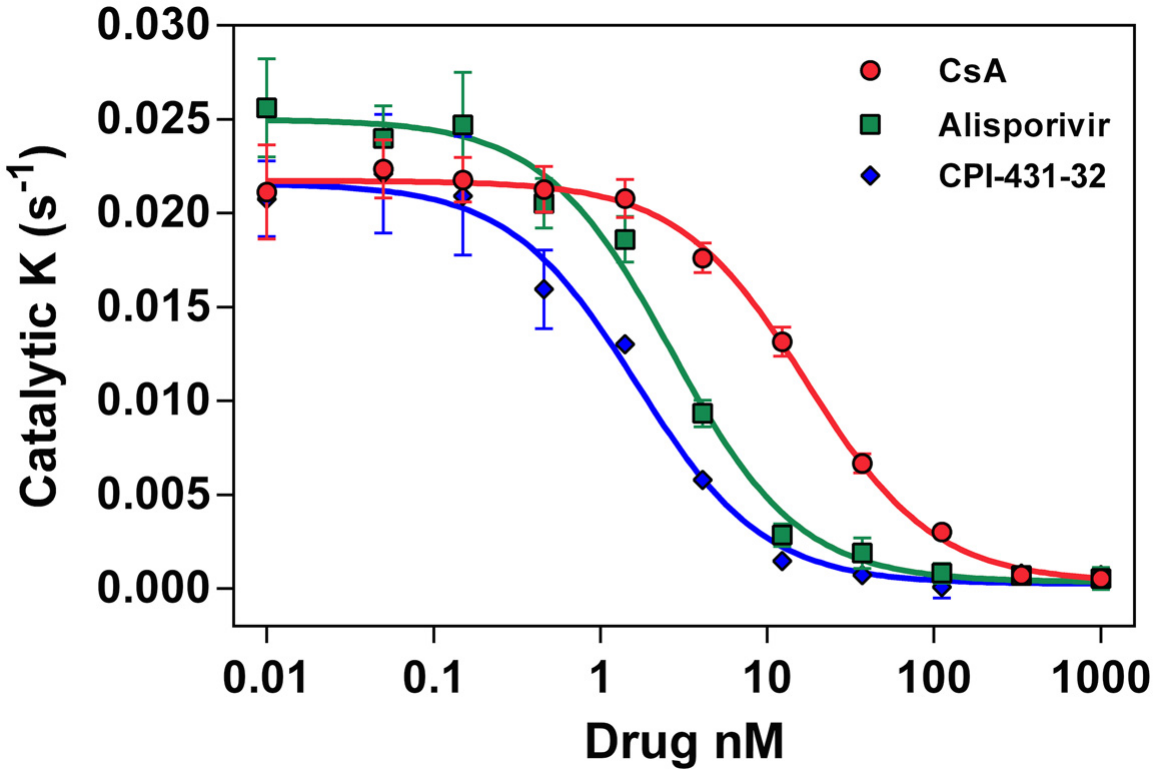
CPI-431-32 inhibits the isomerase activity of CypA. CypA inhibition was assessed with the chymotrypsin-coupled isomerase inhibition assay. Representative data from one experiment is shown. Each symbol is the mean (± SE) of 4 replicate enzyme reactions. Mean IC_50_ values were 1.8 ± 0.6 nM (mean ± SD), 2.8 ± 0.4 nM, and 16.8 ± 2.3 nM for CPI-431-32, ALV and CsA, respectively.

To determine if the relative potencies of CPI-43-32 and ALV in the above experiments are related to their ability to inhibit CypA, we assayed their activities in the chymotrypsin-coupled CypA isomerase assay based on the original version developed by Fischer et al. [56]. CypA inhibition was assessed with 10 concentrations of each compound to determine IC_50_ values. The results paralleled other data in this report in that CPI-431-32 demonstrated the highest potency of CypA inhibition, followed closely by ALV and more distantly by CsA. Three separate experiments all demonstrated statistically significant differences in mean IC_50_ between the compounds (unpaired t-tests, p<0.05). In one representative experiment IC_50_ values were 1.8 ± 0.6 nM (mean ± SD), 2.8 ± 0.4 nM, and 16.8 ± 2.3 nM for CPI-431-32, ALV and CsA, respectively (Fig 3).

### CPI-431-32 blocks HIV-1 reverse transcription and nuclear import

**Fig 4:**
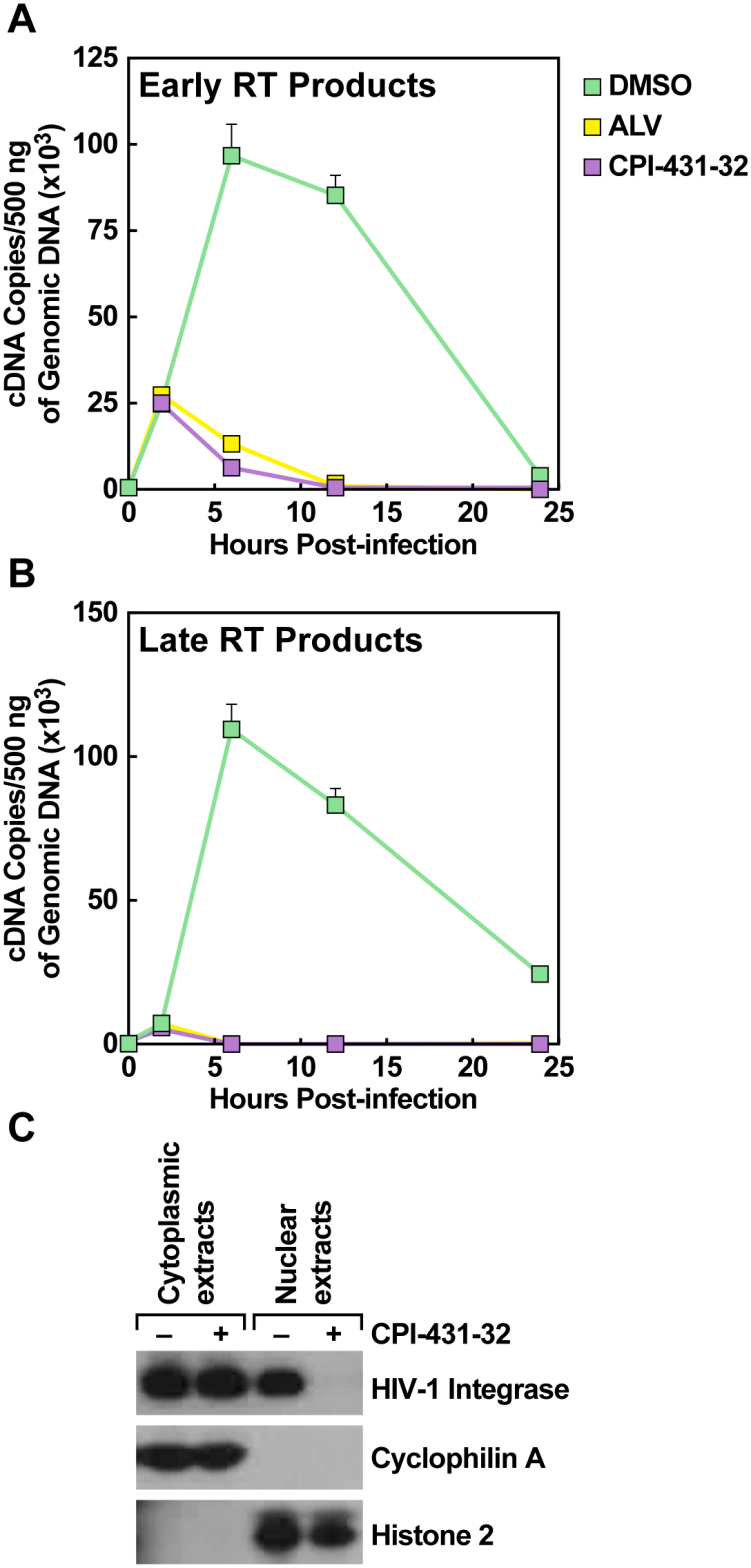
CPI-431-32 blocks HIV-1 reverse transcription and nuclear import. TZM cells were infected with DNase-treated NL4-3 HIV-1 together with DMSO or CPI-431-32 (2 μM). Target cell DNA was isolated at the indicated times and was used to detect early (**A**) and late HIV-1 reverse transcripts (**B**). TZM cells were infected with NL4.3 in the presence or absence of CPI-431-32 (2 μM). Six hours post-infection, cells were fractionated and cytoplasmic and nuclear extracts analyzed by Western blotting (**C**). Results are representative of two independent experiments.

Recent work suggests that the CypA-HIV-1 capsid interaction plays a critical role in the capsid uncoating process which is necessary for HIV-1 reverse transcription, nuclear entry, and integration into the host genome [24]. To examine these early stages of infection in more detail, we first monitored reverse transcription of NL4-3 wild-type HIV-1 in TZM cells in the absence and presence of CypI. DNA was extracted at 2, 6, 12, and 24 h post-infection. Early reverse transcripts (a hallmark of cell entry) and late reverse transcripts (a hallmark of complete reverse transcription) were amplified by PCR with specific pairs of primers. At two hours post-infection, amounts of early and late reverse transcripts were similar in DMSO- and CypI-treated cells, suggesting that CypI did not affect HIV-1 entry into cells nor the earliest stages of reverse transcription (Fig 4A and 4B). However, at six hours post-infection and beyond, early and late RT products were very much lower in CypI-treated cells, suggesting impairment in reverse transcription or degradation of newly made cDNA. CypI-treated cells that did not contain late reverse transcripts also did not contain long terminal repeat circles, a hallmark of nuclear import (data not shown). These results are in accordance with the hypothesis that CypA plays a critical role in reverse transcription.

To further elucidate the effects of CypA-capsid disruption, we analyzed how CPI-431-32 affects the distribution of incoming HIV-1 cores within the first 6 hours of infection as determined by the presence of the HIV-1 integrase protein. TZM cells were infected with HIV-1 NL4.3 for 6 h in the absence or presence of CPI-431-32, then fractionated into cytosolic and nuclear extracts for quantitation of HIV-1 integrase by Western blotting. Purity of the fractions was confirmed by identifying CypA exclusively in cytosolic extracts and histone 2 exclusively in nuclear extracts. Similar amounts of HIV-1 integrase were found in the cytosol of DMSO-treated and CPI-431-32-treated cells, which is consistent with our previous interpretation that cyclophilin inhibition does not affect viral entry into cells (Fig 4C). In contrast, we found that CPI-431-32 completely prevented the nuclear accumulation of integrase. Together these results strongly suggest that CPI-431-32 inhibited HIV-1 infection by interfering with reverse transcription and nuclear import of the viral integration complex.

### CPI-431-32 blocks HCV-mediated double-membrane vesicle (DMV) formation

**Fig 5:**
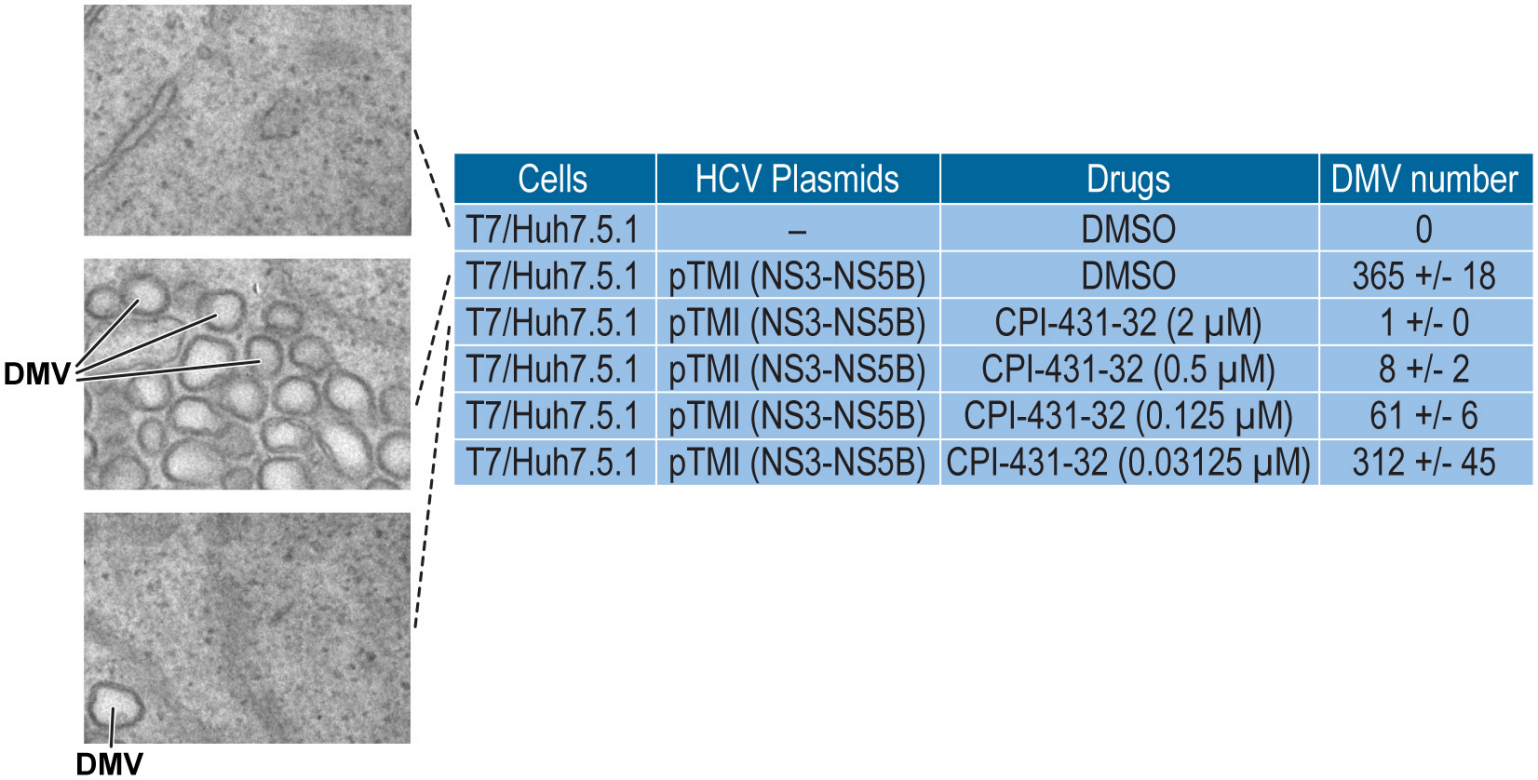
CPI-431-32 blocks HCV-mediated double-membrane vesicle (DMV) formation. T7 polymerase expressing Huh7.5.1 cells were transfected in triplicates with JFH-1 NS3-NS5B plasmid in the presence of DMSO or increasing concentrations of CPI-431-32 and analyzed by EM as well as by Image J and ITEM software for DMVs quantification as described previously [34]. Illustrating EM images of DMVs are presented with an amplification of 25,000x. Data are representative of two independent experiments.

We then investigated the mechanism by which CPI-431-32 inhibits HCV replication. HCV is well known for its ability to reshape intracellular membranes to optimize RNA synthesis, especially by re-organizing the ER membrane close to the nucleus—the so-called membranous web (MW) [67–68]. More recently, the Bartenschlager lab elegantly demonstrated that HCV creates a large number of organelles in the MW called double membrane vesicles (DMVs) [69], which contain all components necessary for efficient viral RNA replication in a protective membranous compartment [70]. We and others recently obtained evidence that the formation of DMVs highly depends on both CypA and HCV NS5A [60, 71, 72]. We thus asked whether CPI-431-32 inhibits HCV replication by preventing the creation of DMVs. To test this hypothesis, we took advantage of a T7 promoter driven JFH-1 NS3-NS5B DNA plasmid (pTM1(NS3-5B)) developed by the Tai lab that permits expression of NS3, NS4A, NS4B, NS5A and NS5B upon HCV polyprotein processing as well as viral RNA synthesis when transfected in T7 RNA polymerase expressing Huh7.5.1 cells (T7/Huh7.5.1 cells) [61]. Importantly, the expression of the HCV NS3-NS5B polyprotein suffices to create both the MV and DMVs independently of viral replication [61]. All controls were presented previously [60]. As we and others recently reported, NS3-5B expression mediates the formation of a significant number of DMVs in DMSO-treated cells (Fig 5). CPI-431-32 profoundly decreased the NS3-NS5B-mediated formation of DMVs compared to DMSO control (Fig 5). These data suggest that CPI-431-32 inhibits HCV replication by preventing the formation of DMVs where viral RNA replication should occur in a protective compartment.

## Discussion

In this study we asked whether a single drug can be used for the simultaneous treatment of two distinct viruses—HIV-1 and HCV. We demonstrated that the cyclophilin inhibitors, CPI-431-32 and ALV, can efficiently block the viruses *in vitro* either as mono-infections or as co-infections in a unique co-culture model. Moreover, we showed that CypI were efficacious towards DAA drug-resistant HIV-1 and HCV variants. CypI were found to block the interactions between CypA and HIV-1 capsid or HCV NS5A, resulting in inhibition of HIV-1 reverse transcription and nuclear import, and inhibition of HCV-induced double membrane vesicles where HCV replication occurs. In all of the assays CPI-431-32 showed higher potency than ALV. Note that the antiviral potencies of CPI-432-31 are not due to general cytotoxicity of the compound, as 3-day incubations with human T cells demonstrated no cytotoxicity by high concentrations (>50 μM) of the compound (data not shown). This lack of cellular toxicity is also true for other CypI including ALV, SCY-635 and NIM811. This is likely due to the fact that CypA is not necessary for cell (CypA-knockout cells) or mice (CypA-knockout mice) viability [73–74].

We report for the first time a novel *in vitro* co-culture system mimicking the prevalent co-infection occurring in HIV-1-infected patients. The co-culture system permits the daily analysis of HIV-1 and HCV replication in a mixed population of infected human CD4+ T-lymphocytes and hepatoma cells. This assay allowed us to identify CypI as potent inhibitors of HIV-1/HCV co-infection. In this co-culture system we observed no substantial influence of one virus on the replication of the other virus. That is, HIV-1 replication was similar in the absence or presence of HCV, and vice versa. At one-week post-HIV-1 infection, large syncytia composed of fused infected CD4+ T-lymphocytes were detected on the surface of adherent hepatoma cells, but this occurred both in the presence or absence of HCV (data not shown). Moreover, no difference in CD4+ T-lymphocytes viability was observed in the presence or absence of HCV (data not shown). This novel *in vitro* co-infection assay will permit the screening of compound libraries for additional drugs that simultaneously inhibit infection of HIV-1 and HCV.

Identifying a compound that blocks the replication of two distinct viruses is not unprecedented. For example, the nucleoside reverse transcriptase inhibitors emtricitabine and lamuvidine potently inhibit both HIV-1 and hepatitis B virus (HBV) infection. They mimic nucleosides but lack a free hydroxyl group at the 3' end, and the reverse transcriptases of HIV-1 or HBV recognize them as regular nucleotides and insert them into the newly synthesized DNA chain. Once inserted, DNA elongation is halted because no further nucleotides can be added due to the lack of the 3' hydroxyl group and the inability to form 5'-3' phosphodiester bonds. This process is called chain termination. Thus, these inhibitors mediate a dual inhibitory effect on HIV-1 and HBV due to the fact that these two viruses use a similar mechanism to synthesize and replicate their genome, and that emtricitabine and lamuvidine mediate a similar block—termination of the viral DNA elongation during the reverse transcription.

CPI-431-32 exerts its major effects by disrupting the interactions of CypA with the viral proteins, HIV-1 p24 capsid and HCV NS5A. In HIV-1 infection, CypA is thought to bind to the capsid core immediately after viral entry into the cytosol of the target cell and helps to stabilize the core during its transport to the nucleus. After docking to the nucleopore via specific components of the nucleopore complex, it is thought that a process of core uncoating takes place which allows passage of the viral genome into the nucleus and ultimately integration into the host DNA [24, 75]. By disrupting the CypA-capsid interaction, CPI-431-32 destabilizes the core and causes premature uncoating and detection of the viral genome by host cell sensors.

CypAhas a similar mode of action in HCV infection, that is the masking of the virus from innate antiviral mechanisms, but later in the HCV life cycle. After cellular entry, HCV RNA is decapsidated and used both for polyprotein translation and RNA replication in the cytoplasm. Translation occurs at rough endoplasmic reticulum (ER) and produces a single polyprotein, which is cleaved by cellular and viral proteases to produce structural and nonstructural proteins. Replication and post-translational processing takes place in the membranous web, which consists of nonstructural proteins and host proteins located at the perinuclear membrane. CypA binds to the membrane-anchored nonstructural protein, NS5A, and triggers the creation of DMVs. In this new membranous compartment, HCV RNA replication occurs in a sheltered manner. In the presence of CPI-431-32, CypA is unable to bind to NS5A and form DMVs. Unprotected viral RNA and proteins are now exposed to cellular defense sensors, leading to an abortive infection. Therefore, the mode of action of CPI-431-32 on HIV-1 and HCV is very similar and is thought to be the removal of the protective “shell” (either core or DMVs) that surrounds the viral genome during the early steps of infection resulting in a vulnerable visibility of the viral genomes to cellular sensors and degradation factors.

The use of CypIs in the clinic is very promising for several reasons. It is generally acknowledged that all future anti-HCV regimens will be composed of a cocktail of several DAAs, which neutralize various viral proteins such as the NS3 protease, the NS5B polymerase and the NS5A protein. By targeting multiple steps of the HCV life cycle, the chances of success for a cure are greatly improved. Thus, CypIs such as CPI-431-32, should be attractive components of future anti-HCV regimens. There are many advantages of host-targeting drugs over direct-acting antivirals that target the virus: higher barrier to resistance, broader coverage of different genotypes/serotypes, and more potential druggable targets. Resistance is a major challenge for DAAs. Indeed, HCV is renown to rapidly mutate the viral targets of DAAs, rendering the antivirals ineffective. CypI have shown promising effects both *in vitro* and in patients to prevent the emergence of resistance and to cure HCV infection either alone or in combination with other agents [76]. Another advantage of using CypI in the clinic is that in contrast to a majority of DAAs, they possess the ability to block the replication of several viruses including HIV-1, coronaviruses, influenza and human papilloma viruses [77]. The ability of CypI to inhibit the dual infection of HIV-1 and HCV represents a significant advantage for the treatment of co-infected patients. In the U.S. alone, 1.4 million patients are infected with HIV-1, 4 million with HCV, and 0.3 million co-infected. Five to ten million patients are co-infected worldwide. Altogether the findings presented in this study and the specific features of CypI make them, especially CPI-431-32, attractive components of an effective antiviral regimen for HIV-1/HCV co-infected patients that would comprise inhibitors of both host and viral targets.
